# Ozone Therapy as a Possible Option in COVID-19 Management

**DOI:** 10.3389/fpubh.2020.00417

**Published:** 2020-08-25

**Authors:** Alessandra Gavazza, Andrea Marchegiani, Giacomo Rossi, Marianno Franzini, Andrea Spaterna, Sara Mangiaterra, Matteo Cerquetella

**Affiliations:** ^1^School of Biosciences and Veterinary Medicine, University of Camerino, Matelica, Italy; ^2^Oxygen-Ozone Therapy Scientific Society (SIOOT), Gorle, Italy; ^3^Director of Comunian Clinic, Gorle, Italy

**Keywords:** COVID-19, ozone, public health, SARS-CoV-2, therapy

The rapid and pandemic outbreak of SARS-CoV-2 causing COVID-19 recognizes in the containment of the infection and in its therapeutic management the two most addressed and challenging topics. Recent guidelines suggest that person-to-person transmission (droplets and aerosol) are the main transmission routes and that, although less likely, also contact with surfaces and objects on which the virus is present can represent a risk (1, 2). With regard to treatment, many clinical trials are ongoing worldwide (3), but no specific antiviral treatment is unanimously recognized leaving to supportive care and symptoms management the most recommended approach (2, 4).

Ozone has extensively been studied in medicine and currently applied at different possible concentrations in various disciplines such as dentistry, dermatology, acute and chronic infectious diseases, and pneumology (5, 6). Chemically it is formed by a triatomic dynamically unstable molecule of oxygen that in gaseous form has a half-life of about 1 h at room temperature, rapidly reverting to oxygen (5). Regarding ozone-related risks, as environmental pollutant it has been shown to reduce maximal transpulmonary pressure, increases respiratory rate and decreased tidal volume as well as significantly increases mean airway resistance and specific airway resistance possibly contributing to increased Influenza A infection (6). Furthermore, it has been shown that the lipid peroxidation operated by high concentration of ozone at the alveolar level can cause strong structural alterations of the surfactant, in a dose and time dependent manner. Strong fusion of lamellar bodies (LBs), associated to the appearance of increasing concentrations of densely coiled LB-like shapes in the alveolar lavage, are resulting ultrastructural changes in type II alveolocites (7). At the same time, it occurs also a strong reduction of organized tubular myelin structures. This is likely due to the fact that medium-high concentration of ozone induce alveolar lesions as consequence of phospholipid peroxidation, causing time-dependent alterations in the organization of stored, and secreted surfactant membranes (8); as a result, administration of gaseous ozone must be avoided.

For medical purposes, ozone can be administered parenterally with minimal side effects, beside the only exception of not being injected intravenously as a gas because of the risk of embolism (5). As a powerful oxidant, when ozone comes into contact with blood or other body fluids, it releases reactive oxygen species (ROS), and lipid oxidation products (LOPs) both of which are responsible for the biological results (5). The main form of ROS is hydrogen peroxide (H_2_O_2_) which is easily transferred from plasma into the cells. When H_2_O_2_ abruptly appears above the threshold medical concentration in the cytoplasm of cells it represents the triggering stimulus for the possibly simultaneous activation of different biochemical pathways in erythrocytes, leukocytes and platelets in addition to other numerous biological effects, such as antimicrobial, immunostimulant, and antioxidant ones. H_2_O_2_ is then suddenly inactivated into water by the high concentration of glutathione (GSH), catalase (CAT), and glutathione peroxidase (GSH-Px) enzymatic systems, reducing its harmful potential (5). Although the exact mechanism of action of ozone is far to be fully elucidated, it has been characterized to have different biological properties. For example, it has been showed to facilitate wound healing by promoting the release of oxygen, platelet-derived growth factor and transforming grow factor β (9). Ozone is also regarded as capable to activate the immune system increasing the production of interferon and interleukin-2 and decreasing tumor necrosis factor (TNF) levels (6). In addition to this, ozone stimulates both the red blood cell glycolysis rate leading to an increased amount of oxygen released to the tissues and the Krebs cycle resulting in an increased production of ATP. It also reduces significantly NADH concentration and helps to oxidize cytochrome C, thus stimulating oxygen metabolism (6), as well as it shows anti-inflammatory and possible cytoprotective action interacting with NF-KB and Nrf2 transcription agents (10, 11). The paradox that ozone exerts an antioxidant response (known as oxidative preconditioning) capable of reversing a chronic oxidative stress is related to the stimulation of production free radical scavengers and cell-wall protectors such as glutathione peroxidase, catalase, and superoxide dismutase (5, 12).

Through the oxidation of double bonds, ozone possesses the unique ability to inactivate biological contaminants, including viruses. Ozone disrupts the integrity of the bacterial cell walls causing their lysis and death (5, 13), and is able to effectively control spore germination of various dermatophytes (14, 15). Data obtained throughout years of research suggest that ozone inactivation of viruses occurs primarily in by lipid and protein peroxidation (16). Lipid peroxidation is initiated by different ROS, including H_2_O_2_. Through oxidation of the unsaturation along the hydrocarbon chain of fatty acid component of phospholipid membrane it causes severe structural and functional damage to the lipid bilayer of the plasma membrane (17). On the other hand, protein peroxidation is due either to interaction of protein with ROS or by interaction with secondary byproducts of oxidative stress; both of them cause irreversible oxidative changes that inhibit normal cellular mechanisms. These include loss of aggregation and proteolysis control, changes in enzyme-substrate binding activities, and modifications in immunogenicity (18). Protein peroxidation particularly seems to play a key role in the inactivation of non-enveloped viruses, such as adenovirus, poliovirus and other enteroviruses (19, 20). Murray and coworkers (21) demonstrated few years ago the efficacy of ozone against a variety of simple and complex viruses, including enveloped, non-enveloped, DNA, and RNA ones. Vesicular stomatitis Indiana virus (VSIV), adenovirus type-2 (HAdV-2), and selected strains of herpes simplex virus type-1 (HHV-1), vaccinia virus (VACV), influenza A virus (FLUAV) pools were exposed *in vitro* to a minimal amount of ozone (from 800 to 1,500 parts per million by volume), and it was effective in inactivating all these viruses. More in detail, enveloped viruses such as VSIV, HHV-1, VACV, and FLUAV showed great sensitivity to ozone while the non-enveloped HAdV-2 was more but not completely resistant to ozone. The results of the study suggest a direct and irreversible damage and destruction of the lipid viral envelope and protein capsid confirming the ability of ozone as a tool for the control of some viruses (21). Ozone therapy has recently been suggested as a possible economic and easily available further option for Sars-CoV-2 (22) thanks to its immunomodulatory, anti-inflammatory and biocide action and to the nitric oxide associated and dependent antiplatelet effect (23, 24). About the relationship between ozone and Sars-CoV-2 is also worth noting the “triangle” existing among human angiotensin-converting enzyme 2 (ACE2), that both is a receptor facilitating virus entry and, as fundamental component of renin-angiotensin system, also protects from acute lung injury, and Nrf2 pathway modulation, influencing ACE2 activity and being in turn influenced by ozone (10, 11, 25–27). Interestingly, the virus has also been found in substrates other than respiratory secretions, such as fecal swabs and blood (4), suggesting a possible interaction with the virus in case ozone is in the blood. Recently, the Italian “Istituto Superiore di Sanità” (National Institute of Health) answering to Prof. Franzini, member of “Scientific Society of Oxygen Ozone Therapy” Directive Board, recognized that oxygen-ozone therapy, after Ethical Committee approval and under patient informed consent, could represent a possible option (28). Remarkably, in this regard, two recent reports of the “Scientific Society of Oxygen Ozone Therapy,” referring to patients affected by COVID-19 undergoing immediately after hospitalization, in addition to standard therapy, also to autohemotherapy with ozonated blood, furnished very encouraging results (29, 30). Moreover, also other reports hypothesizing the use of ozone in COVID-19 are being progressively undertaken and published (31, 32).

Gas concentration, route of administration, safety, stage of the disease in which administer it, patients' selection, contraindications, concomitant administration of antioxidants, etc., are some of the aspects that need to be further addressed with regard to its eventual use in COVD-19 patients, but in the authors opinion ozone therapy is an option that could deserve to be explored while waiting for specific treatments and for a vaccine.

## Author Contributions

All authors listed have made a substantial, direct and intellectual contribution to the work, and approved it for publication.

## Conflict of Interest

MF is Director of Comunian Clinic, Gorle (BG) where ozone therapy is routinely practiced. The remaining authors declare that the research was conducted in the absence of any commercial or financial relationships that could be construed as a potential conflict of interest.
